# A 1:1 matched-pair comparative study of minimally invasive elastic Poller wire augmentation vs. standard augmentative plating for hypertrophic femoral non-union after intramedullary nailing

**DOI:** 10.3389/fmed.2026.1877644

**Published:** 2026-08-11

**Authors:** Jinxian Zhao, Zeqian Zhang, Jianhui Lin, Guokai Feng, Zongquan Mo, Yongqiang Lao, Shuping Yu

**Affiliations:** 1 The Eighth Clinical Medical College of Guangzhou University of Chinese Medicine Province, Guangdong, China; 2 Foshan Hospital of Traditional Chinese Medicine Province, Guangdong, China

**Keywords:** augmentative plating, biological osteosynthesis, femoral non-union, hypertrophic, intramedullary nail, poller wire

## Abstract

**Background:**

The primary cause of hypertrophic non-union following femoral intramedullary nailing (IMN) is mechanical instability rather than the depletion of biological activity. Although standard augmentative plating can provide excellent stability, it typically necessitates extensive surgical exposure and periosteal stripping, which disrupts the fragile local vascular network. This study aimed to introduce a novel, minimally invasive elastic Poller wire technique and compare its clinical efficacy and perioperative surgical trauma with those of standard augmentative plating.

**Methods:**

This retrospective cohort study included 32 patients with hypertrophic femoral non-union with a retained IMN treated between 2017 and 2025. To control for selection bias, a 1:1 matched-pair design was utilized. The patients were assigned to two groups based on the surgical intervention: the Poller wire group (*n* = 16) underwent the minimally invasive elastic Poller wire technique; the plate group (*n* = 16) consisted of patients who underwent standard open augmentative plating, strictly matched to the Poller wire group based on baseline characteristics including age, gender, and time to non-union. The primary outcome measures were the radiographic bone union rate and time to union. Secondary outcomes included operative time, intraoperative blood loss, incision length, and the incidence of complications.

**Results:**

All 32 patients (100%) successfully achieved solid bone union. There was no significant difference in the mean time to union between the plate and Poller wire groups (5.1 ± 1.2 vs. 4.9 ± 1.1 months, *P* = 0.584). However, the Poller wire group demonstrated significant advantages in perioperative parameters, including a shorter incision length (4.2 ± 0.8 vs. 16.5 ± 2.4 cm, *P* < 0.001), shorter operative time (42.3 ± 8.5 vs. 118.5 ± 15.6 min, *P* < 0.001), and substantially reduced intraoperative blood loss (45.0 ± 12.5 vs. 350.5 ± 65.2 ml, *P* < 0.001). Furthermore, all patients in the plate group required inpatient admission for a secondary major surgery for implant removal, whereas all implants in the Poller wire group were successfully removed via a minimally invasive approach under local anesthesia.

**Conclusion:**

For hypertrophic femoral non-union, the minimally invasive elastic Poller wire technique provides adequate mechanical stability, achieving a 100% union rate comparable to the gold standard of augmentative plating. By maximally preserving the biological healing envelope and significantly reducing surgical trauma and medical costs, this technique represents a highly promising minimally invasive alternative in clinical practice.

## 1 Introduction

Reamed intramedullary nailing (IMN) is considered the “gold standard” for the treatment of femoral shaft fractures. However, the incidence of post-operative non-union can still reach up to 10%. Based on biological characteristics, hypertrophic non-union is characterized by the abundant formation of an “elephant foot” callus. This robust biological response indicates that the fracture ends possess strong osteogenic potential; the fundamental obstacle to union is the lack of adequate mechanical stability. Typically, this instability is caused by an excessively widened medullary canal, which leads to a “windshield wiper effect” or excessive rotational micromotion of the IMN within the canal (1).

According to the “Diamond Concept” of fracture healing, the management of hypertrophic non-union should prioritize the optimization of the mechanical environment rather than biological intervention. Retaining the IMN and applying a single augmentative plate is a widely recognized and effective strategy associated with extremely high union rates (2). However, as a form of “maximal fixation,” augmentative plating inevitably requires an extensive incision, massive periosteal stripping, and the dissection of deep musculature. This not only results in significant surgical trauma and blood loss but also destroys the fragile neovascular network surrounding the non-union site, which contradicts the core principles of modern biological osteosynthesis (3).

The principle of Poller screws (blocking screws) has been proven effective in reducing the effective diameter of the medullary canal and enhancing the mechanical stiffness of the IMN construct. Inspired by this (4), we hypothesized that the use of multiple Kirschner wires (K-wires) could generate a similar blocking effect. Through a designated mini-incision approach, we attempted to insert K-wires adjacent to the IMN to serve as “elastic Poller wires,” aiming to provide adequate, adjustable stability to awaken the healing process while achieving ultimate protection of the soft tissue envelope. This study utilized a rigorous 1:1 matched-pair design to compare the clinical outcomes of this novel minimally invasive technique with those of standard open augmentative plating (5, 6).

## 2 Materials and methods

### 2.1 Patient selection and 1:1 matching design

This retrospective study was approved by the Institutional Review Board of our hospital. Data from patients who underwent revision surgery for femoral non-union following IMN at our department between 2017 and 2025 were collected.

Inclusion criteria: (1) Diagnosis of aseptic femoral shaft non-union (>9 months post-injury, or no radiographic progression of healing for three consecutive months); (2) Radiographic evidence of hypertrophic or oligotrophic non-union (presence of callus); (3) Retention of the primary IMN.

Exclusion criteria: (1) Atrophic non-union; (2) Active infection; (3) Implant failure (e.g., broken nail); (4) Pathological fractures.

1:1 matching process: following strict screening, 16 patients treated with the minimally invasive elastic Poller wire technique were included as the Poller wire group (Group B). To eliminate potential confounding variables, we strictly matched these patients 1:1 with those from our institutional database who underwent standard augmentative plating during the same period. The matching priorities were: (1) Non-union type (all hypertrophic); (2) Identical gender; (3) Age difference ≤ ±5 years; (4) Similar interval from the initial surgery to revision. Ultimately, 16 matched patients were assigned to the plate group (Group A). A total of 32 patients were included in this study.

The clinical choice between plate augmentation and the elastic Poller wire technique was primarily influenced by the chronological evolution of our clinical practice and surgeon preference. Standard augmentative plating was primarily utilized during the earlier period of this cohort (2017–2021). As our department actively sought more minimally invasive options to reduce surgical trauma, the elastic Poller wire technique was developed and increasingly adopted from 2021 onwards. To mitigate potential confounding beyond the matched variables, baseline comorbidities (such as diabetes, hypertension, and smoking status) were thoroughly reviewed from medical records, demonstrating no significant imbalances between the two groups that could significantly skew the observed treatment effect size.

### 2.2 Surgical technique

**Figure 1:**
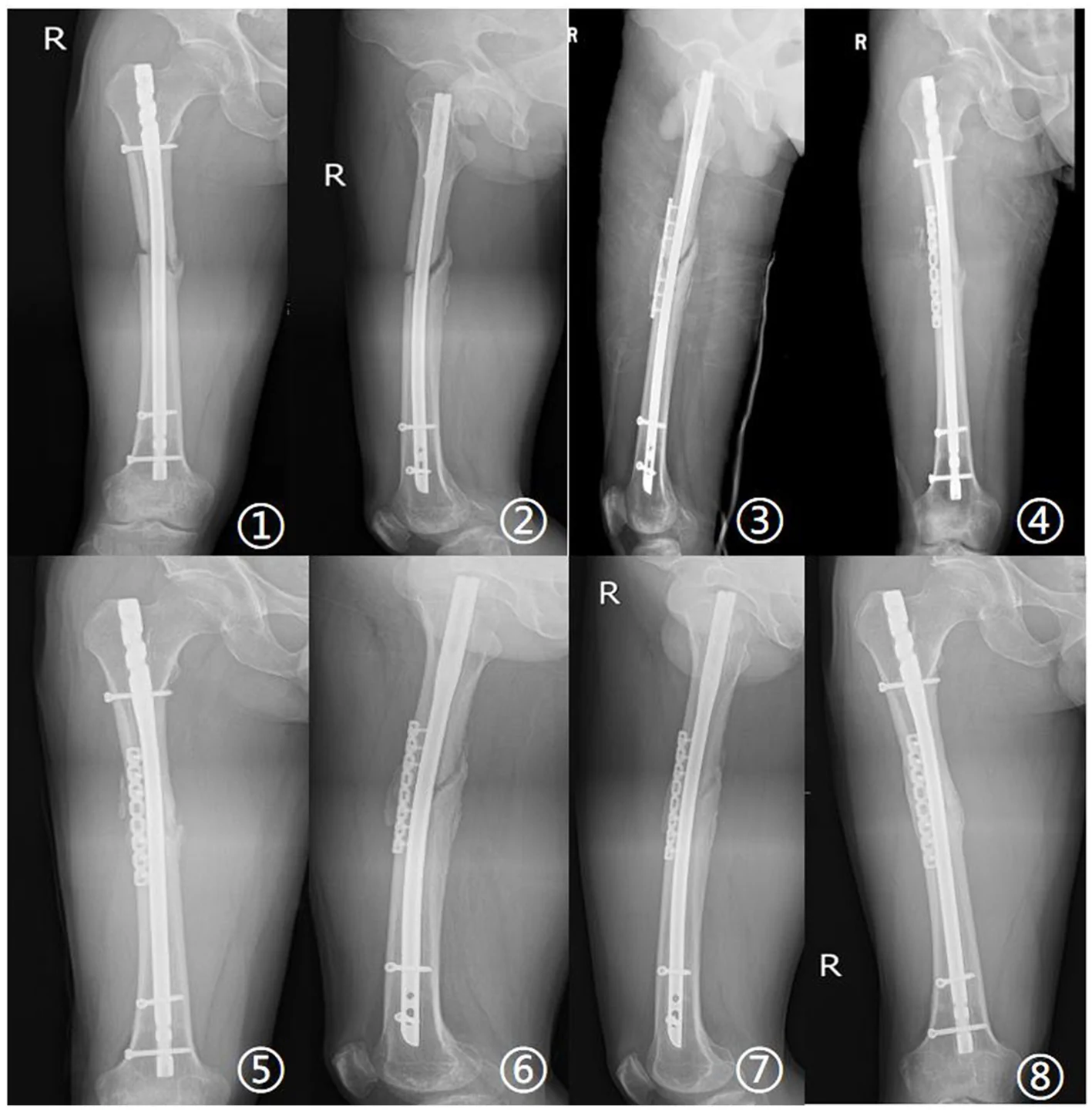
Standard open augmentative plating for hypertrophic femoral non-union. **(1, 2)** Pre-operative anteroposterior (AP) and lateral radiographs showing a typical hypertrophic non-union after reamed femoral IMN. An abundant “elephant foot” callus is visible at the fracture ends, indicating an active biological response that failed to bridge due to mechanical instability. **(3, 4)** Immediate post-operative AP and lateral radiographs. An extensive lateral approach was used to apply a lateral locking compression plate. This “maximal fixation” strategy provided absolute stability at the expense of extensive soft tissue stripping. **(5, 6)** Follow-up radiographs at 3 months showing gradual callus remodeling and bridging across the fracture line. **(7, 8)** Radiographs at 6 months showing the complete disappearance of the fracture line, achieving solid bone union.

Plate group: a standard lateral femoral approach (typically >15 cm in length) was utilized. The iliotibial tract was incised, and the vastus lateralis was extensively elevated to expose the non-union site. Following the debridement of soft tissues at the fracture ends, a locking compression plate was applied to the lateral cortex, spanning the fracture line. Under fluoroscopic guidance to avoid the retained IMN, at least three bicortical screws were inserted into both the proximal and distal segments Figure 1.

**Figure 2:**
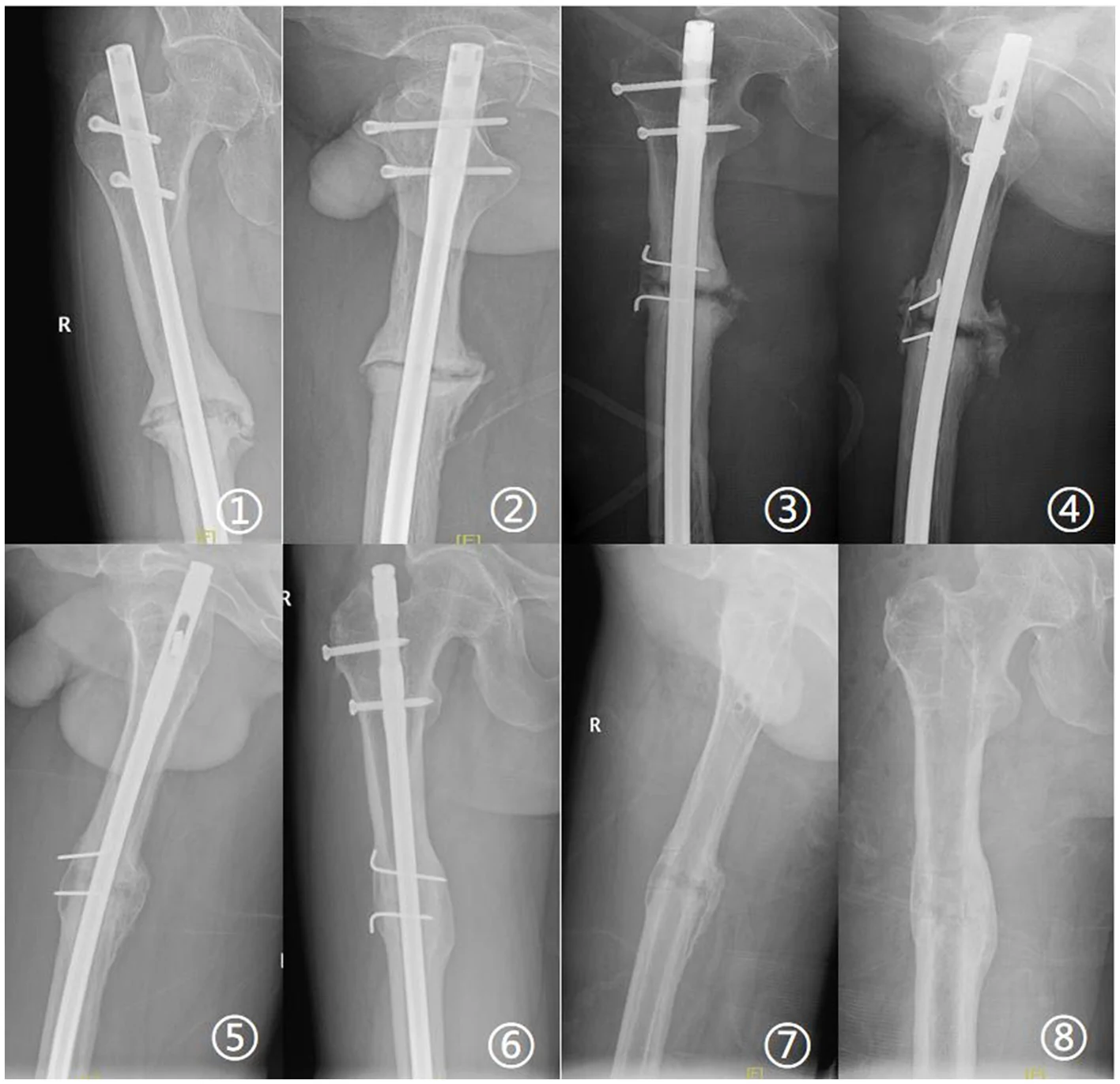
Minimally invasive elastic Poller wire augmentation for hypertrophic femoral non-union. **(1, 2)** Pre-operative AP and lateral radiographs showing hypertrophic non-union. The IMN remains intact, but sclerotic margins and abundant callus are present due to micromotion. **(3, 4)** Immediate post-operative radiographs. Two 2.5 mm K-wires were inserted transcortically via a 3–5 cm mini-incision. The wires lay tightly against the IMN and penetrated both cortices, acting as wedges to eliminate the micromotion (“windshield wiper effect”) of the IMN. The wire tails were bent to prevent intra-medullary migration and to facilitate future minimally invasive removal. **(5, 6)** Radiographs at 3 months showing an improved stress environment with massive bridging callus. **(7, 8)** Radiographs at 6 months showing complete union and partial recanalization of the medullary cavity after the K-wires were removed via a minimally invasive approach under local anesthesia, demonstrating excellent biological preservation.

**Figure 3:**
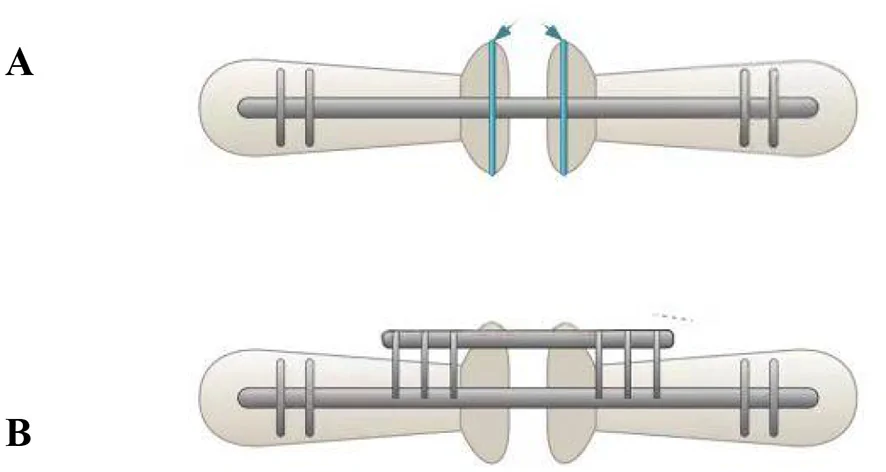
Schematic diagram illustrating the biomechanical and biological comparison of the two augmentation strategies. **(A)** Minimally invasive elastic Poller wire technique: transcortical K-wires (blue) are inserted adjacent to the retained IMN through a mini-incision. These wires act as elastic wedges to abolish deleterious rotational and shear micromotion while preserving a minimal physiological axial strain to stimulate the conversion of soft callus into hard bone. The periosteal and soft tissue envelope remains largely undisturbed. **(B)** Standard augmentative plating: a long lateral locking compression plate is applied to achieve rigid fixation (absolute stability). However, this maximal fixation requires an extensive lateral approach, resulting in severe disruption of the periosteum and the local osteogenic soft tissue envelope (red dashed area).

Poller wire group: under multi-planar fluoroscopic guidance (anteroposterior and lateral views), a mini-incision measuring approximately 3–5 cm was created, centered precisely over the hypertrophic non-union site Figure 2. Soft tissues were bluntly dissected down to the bone cortical surface, strictly avoiding periosteal stripping. In all cases, the primary, intact intramedullary nail (IMN) was intentionally retained. To abolish the “windshield wiper effect,” two to three stout Kirschner wires (K-wires, 2.5 mm in diameter) were transcortically inserted into the widened segment of the medullary canal under direct vision and continuous image intensifier control. These wires were driven from the lateral or anterolateral cortex, passing immediately adjacent to and tightly abutting the surface of the retained IMN to act as rigid mechanical “wedges.” The wires crossed the non-union line or were placed within 2 cm proximal and distal to it, ultimately penetrating the contralateral cortex to achieve secure bicortical purchase. Intraoperative mechanical stability was manually confirmed by applying rigorous rotational and bending stresses to the femur, verifying the absence of gross micromotion at the non-union site under real-time fluoroscopy. Finally, the extraosseous tail ends of the K-wires were bent into a “*U*” shape to prevent migration, cut short, and buried deeply beneath the deep fascia Figure 3.

### 2.3 Post-operative management and outcome evaluation

Patients in both groups were encouraged to commence range-of-motion exercises immediately post-operatively. Partial weight-bearing with crutches was permitted 2 weeks post-surgery. Full weight-bearing was allowed once bridging callus was radiographically evident. In the Poller wire group, the K-wires were routinely removed via the original mini-incision under local anesthesia after solid bone union was confirmed radiographically.

The primary outcome measures were the radiographic union rate and time to union. Secondary outcomes included operative time, intraoperative blood loss, incision length, length of hospital stay, and complications such as infection, implant irritation, and the rate of secondary surgical intervention.

### 2.4 Statistical analysis

Statistical analyses were performed using SPSS version 27.0. To properly account for the 1:1 matched-pair design and control for intra-pair correlation, the paired samples *t*-test was utilized to analyze differences in continuous variables (presented as mean ± standard deviation) between the two matched groups. For categorical variables, the McNemar test was applied to evaluate discordant pairs. A *P*-value of < 0.05 was considered statistically significant. Furthermore, due to the small sample size and to avoid inappropriately interpreting non-significant *p*-values as definitive therapeutic equivalence, a *post-hoc* statistical power analysis was conducted for the primary outcome (time to union) using G^*^Power software (version 3.1.9.7).

## 3 Results

### 3.1 Baseline characteristics

Through rigorous 1:1 matching, the baseline characteristics of the 32 patients in both groups were highly balanced. The mean age was 43.5 ± 11.2 years in the plate group and 42.1 ± 10.8 years in the Poller wire group (*P* = 0.685). The two groups exhibited excellent comparability in gender distribution, BMI, and time from initial surgery to revision (all *P* > 0.05; Table 1).

**Table 1:** Comparison of baseline characteristics.

Parameter	Plate group (*n* = 16)	Poller wire group (*n* = 16)	*P*-value
Age	43.51 ± 1.2	42.11 ± 0.8	0.685
Gender (male/female)	10/6	10/6	1.000
BMI (kg/m^2^)	25.12 ± 0.9	24.83 ± 0.1	0.756
Time to revision (months)	10.52 ± 0.1	11.02 ± 0.4	0.487
Hypertrophic non-union (*n*)	16	16	1.000

### 3.2 Perioperative parameters

The Poller wire group demonstrated significant advantages in minimizing surgical trauma. The incision length in the Poller wire group was significantly shorter than that in the plate group (4.2 ± 0.8 vs. 16.5 ± 2.4 cm, *P* < 0.001). Consequently, the operative time (42.3 ± 8.5 vs. 118.5 ± 15.6 min, *P* < 0.001) and intraoperative blood loss (45.0 ± 12.5 vs. 350.5 ± 65.2 ml, *P* < 0.001) were substantially reduced in the Poller wire group. The length of hospital stay was also significantly shorter in the Poller wire group (4.5 ± 1.2 vs. 8.5 ± 2.1 days, *P* < 0.001; Table 2).

**Table 2:** Comparison of perioperative and clinical outcome.

Parameter	Plate group (*n* = 16)	Poller wire group (*n* = 16)	*P*-value
Incision length	16.52 ± 0.4	4.20 ± 0.8	<0.001
Operative time	118.51 ± 5.6	42.38 ± 0.5	<0.001
Intraoperative blood loss	350.56 ± 5.2	45.01 ± 2.5	<0.001
Hospital stay	8.52 ± 0.1	4.51 ± 0.2	<0.001
Time to union	5.11 ± 0.2	4.91 ± 0.1	0.584
Final union rate	16 (100%)	16 (100%)	>0.999

*P*-values were calculated using the paired samples *t*-test for continuous variables and the McNemar test for categorical variables.

### 3.3 Clinical and radiographic outcomes

All 32 patients (100%) successfully achieved solid bone union. Complete follow-up was obtained for all patients, with a mean follow-up duration of 12 ± 2.6 months. The mean radiographic time to union was 5.1 ± 1.2 months in the plate group and 4.9 ± 1.1 months in the Poller wire group, indicating no statistical difference (*P* = 0.584). Regarding perioperative safety and complications, the clinical findings were structured and evaluated across specific risk categories: (1) Infection: no cases of superficial or deep surgical site infections were identified in either group. (2) Implant failure: there were no incidents of K-wire migration, backing out, or breakage in the Poller wire group, and no plate or screw breakages occurred in the plate group. (3) Malalignment: post-operative radiographs confirmed acceptable alignment in all 32 patients, with no secondary loss of reduction or malalignment. (4) Neurovascular injury: no iatrogenic nerve palsies or major vascular lacerations occurred during or after the surgical approaches. (5) Delayed Union or Persistent Non-union: all patients achieved solid bone bridging; no persistent non-union or delayed healing beyond expected parameters was recorded. (6) Reoperation: in the plate group, 100% of the patients (16/16) required a secondary major inpatient surgery under regional/general anesthesia for plate removal due to symptomatic hardware prominence. Conversely, in the Poller wire group, no patients required major reoperation, though one patient experienced mild muscle irritation from a wire tail, and all 16 patients successfully underwent elective implant removal via the original mini-incision under local anesthesia (7).

## 4 Discussion

Hypertrophic non-union accounts for a significant proportion of non-unions following reamed IMN of femoral shaft fractures. The underlying pathology is not a deficiency of osteoblasts or growth factors, but rather a high-strain mechanical environment that inhibits the calcification and bridging of the soft callus into hard bone (8). As articulated by the “Diamond Concept” of fracture healing, the core principle in treating hypertrophic non-union is to enhance mechanical stability, typically without the need for additional biological interventions (such as isolated bone grafting) (9). Currently, the standard surgical strategies to resolve this mechanical failure primarily include exchanging for a larger-diameter nail (exchange nailing) and retaining the IMN with augmentative plating.

In mainstream international revision strategies, both “gold standard” procedures possess respective merits and drawbacks. Exchange nailing has the advantage of being performed via a closed approach with a relatively small incision. However, its disadvantages are glaring: it incurs significant intraoperative blood loss, the repeated reaming causes secondary damage to endosteal perfusion, and in patients with an overly capacious medullary canal, even the largest-diameter nail may fail to eliminate micromotion, leading to unpredictable union rates. Conversely (10), augmentative plating neutralizes rotational and shear forces, providing “absolute stability” and yielding extremely high clinical union rates. Nevertheless, its primary limitation is that it constitutes “over-treatment” via “maximal fixation”: it requires a massive incision (often >15 cm) and extensive periosteal stripping, which severely devastates the precious osteogenic soft tissue envelope surrounding the hypertrophic non-union. To seek an optimal balance between “eliminating micromotion” and “preserving blood supply,” we proposed a novel hypothesis: utilizing the “Poller (blocking)” principle by inserting K-wires through a mini-incision to provide adequate mechanical stability at a minimal biological cost (11).

The results of this study confirmed our hypothesis: the minimally invasive elastic Poller wire technique achieved a 100% union rate identical to that of standard plating, while demonstrating significant minimally invasive advantages in controlling surgical trauma (12). The significant reductions in incision length (4.2 vs. 16.5 cm), operative time (42.3 vs. 118.5 min), and blood loss (45.0 vs. 350.5 ml) observed in the Poller wire group carry profound clinical meaningfulness that extends far beyond mere statistical significance. Minimizing intraoperative blood loss effectively mitigates the risks and medical costs associated with allogeneic blood transfusions (13). Furthermore, a substantially shortened operative time minimizes the duration of anesthesia, which reduces the incidence of systemic thromboembolic or pulmonary complications—an advantage of critical importance for elderly or medically fragile patients with multiple comorbidities. Finally, a shorter incision minimizes extensive dissection of the vastus lateralis, thereby facilitating early, accelerated functional rehabilitation and reducing post-operative donor-site pain. Technically, the adoption of a 3–5 cm mini-incision approach permitted safe operation under direct vision, effectively balancing minimal invasiveness with neurovascular protection. The inserted K-wires occupied the space between the IMN and the bone cortex, effectively abolishing the “pendulum effect” (windshield wiper effect). Its greatest theoretical advantage lies in the fact that, unlike a rigid locking plate, the K-wires provide “elastic/relative stability.” This construct permits physiologically negligible axial micromotion at the fracture site, providing the optimal mechanical environment to stimulate the conversion of soft callus to hard bone. Furthermore, this technique offers tremendous health economic value. Compared to the costly plates that often necessitate a major secondary inpatient surgery for removal, the implants in all patients from the Poller wire group were removed non-invasively under local anesthesia in the outpatient clinic, completely relieving patients from the burden of secondary hospitalization. A potential drawback is the risk of K-wire backing out or migration; however, we effectively mitigated this risk by bending the wire tails and burying them deeply beneath the fascia during surgery (14).

This study possesses several distinct limitations that warrant careful consideration. First, its retrospective nature carries inherent historical selection and reporting biases, although a rigorous 1:1 matched-pair design was enacted to balance key baseline characteristics (15). Second, the sample size is notably small (*n* = 32, with 16 pairs), which restricts the statistical power to definitively conclude therapeutic equivalence or detect rare, subtle differences in secondary outcomes. A *post-hoc* power analysis indicated that the study was underpowered to detect a small true difference in the time to union, meaning the non-significant *P*-value (*P* = 0.584) should be interpreted as a lack of evidence of a difference rather than definitive proof of equivalence. This small sample size also limits the generalizability and strength of our safety and complication conclusions. Third, the current study lacks comprehensive, validated functional outcome measures—such as standardized Visual Analog Scale (VAS) pain scores, objective gait analyses, return-to-work timelines, or multi-dimensional quality-of-life indices (e.g., SF-36)—precluding a thorough evaluation of long-term functional recovery. Fourth, the mean follow-up duration of 12 months is relatively short, capturing only early to mid-term outcomes. Finally, the study lacks direct, controlled *in vitro* biomechanical validation to precisely quantify the bending and torsional stiffness of the customized K-wire/IMN composite construct. Multi-center, prospective randomized controlled trials with expanded cohorts and rigorous functional scoring are required to establish long-term validation (16–18).

## 5 Conclusion

In conclusion, this preliminary 1:1 matched cohort study suggests that the minimally invasive elastic Poller wire augmentation technique represents a safe, viable, and highly cost-efficient surgical alternative for the treatment of hypertrophic femoral non-union with a retained intramedullary nail. By providing acceptable mechanical stability while successfully preserving the periosteal biological envelope, this technique achieved promising short-term bone union rates comparable to the standard augmentative plating approach, while substantially mitigating perioperative surgical trauma and eliminating the need for major secondary implant removal surgeries. Given the small sample size and retrospective design of this study, these findings should be interpreted with caution. Nevertheless, the elastic Poller wire approach provides a compelling, minimally invasive treatment option that warrants further evaluation in larger prospective trials.

## Data Availability

The original contributions presented in the study are included in the article/supplementary material, further inquiries can be directed to the corresponding author.
